# The Role of Intestinal Microbiota in Acute Graft-versus-Host Disease

**DOI:** 10.1155/2015/145859

**Published:** 2015-05-18

**Authors:** Yuanyuan Chen, Ye Zhao, Qiao Cheng, Depei Wu, Haiyan Liu

**Affiliations:** ^1^Laboratory of Cellular and Molecular Tumor Immunology, Jiangsu Key Laboratory of Infection and Immunity, Institutes of Biology and Medical Sciences, Soochow University, Suzhou 215123, China; ^2^Cyrus Tang Hematology Center, Department of Hematology, Jiangsu Institute of Hematology, Collaborative Innovation Center of Hematology, The First Affiliated Hospital of Soochow University, Suzhou 215006, China

## Abstract

The mammalian intestinal microbiota is a complex ecosystem that plays an important role in host immune responses. Recent studies have demonstrated that alterations in intestinal microbiota composition are linked to multiple inflammatory diseases in humans, including acute graft-versus-host disease (aGVHD). aGVHD is one of the major obstacles in allogeneic hematopoietic stem cell transplantation (allo-HSCT), characterized by tissue damage in the gastrointestinal (GI) tract, liver, lung, and skin. Here, we review the current understanding of the role of intestinal microbiota in the control of immune responses during aGVHD. Additionally, the possibility of using probiotic strains for potential treatment or prevention of aGVHD will be discussed.

## 1. Introduction

The mammalian gastrointestinal (GI) tract harbors a dense and diverse microbial community, which is composed primarily of bacteria but also includes fungi, archaea, and viruses; collectively, these are referred to as the intestinal microbiota [1]. These microorganisms establish symbiotic relationships with their hosts, playing crucial roles in the digestion of food and exerting a considerable influence on the physiological, metabolic, nutritional, and immunological state of the host [2–4]. Recent studies have demonstrated that alterations in the composition of intestinal microbiota are linked to multiple metabolic and inflammatory diseases in humans, including obesity, inflammatory bowel disease (IBD), colorectal cancer, allergy, type 2 diabetes, liver cirrhosis, rheumatoid arthritis, and neurodevelopmental disorders [5–12]. These associations raise fundamental questions regarding the immunomodulatory mechanisms by which components of the intestinal microbiota and their metabolites influence resistance or susceptibility to a broad range of clinically important diseases.

Allogeneic hematopoietic stem cell transplantation (allo-HSCT) is the only curative therapy for hematologic malignant tumors, bone marrow failure, and congenital metabolic disorders. However, the development of acute graft-versus-host disease (aGVHD) limits the success of allo-HSCT and is fatal to approximately 15% of transplant recipients [13, 14]. aGVHD results from an immunological attack on target recipient organs and tissues (such as the skin, liver, lung, and GI tract) by donor allogeneic T cells that are transferred along with the allograft. The development and severity of aGVHD in transplant recipients depend on factors such as recipient age, toxicity of the conditioning regimen, hematopoietic graft source, and aGVHD prophylaxis approaches [15]. Steroids are the first line of treatment, but patients with steroid-refractory aGVHD have a dismal outcome, with long-term mortality rates that can reach 90% [16]. Recent studies have demonstrated a close relationship between intestinal microbiota composition and the severity of aGVHD [17–19].

## 2. Altered Intestinal Microbiota Composition and Diversity Associated with aGVHD

For many years, our understanding of the composition of mammalian intestinal microbiota depended on culturing and identifying commensal microorganisms. However, this approach is insufficient to catalog intestinal microbial species because the majority of intestinal bacteria cannot be cultured by currently available methods [20]. The emergence of new molecular profiling techniques, such as 16S rRNA sequence-based microbial identification and high-throughput sequencing analysis, has led to a revolution in the understanding of the intestinal microbiota by allowing culture-independent analysis of microbial community composition [21].

The human gut harbors approximately one hundred trillion microbes, which is ten times the number of human cells in the body [22]. Their combined genomes contain more than five million genes, outnumbering the human genetic potential by two orders of magnitude [23]. Several phyla of bacteria constitute the bulk of the human intestinal microbiota. The most abundant phyla in the human intestine are Firmicutes and Bacteroidetes, which constitute over 90% of the human intestinal microbes. The Firmicutes consist primarily of bacteria belonging to the Clostridia class and include a subset of Bacilli (Bacillaceae, Enterococcaceae, and Lactobacillaceae), which are capable of oxidizing organic sugars via fermentation to produce large amounts of lactic acid and carbon dioxide [24, 25]. Members of the gut bacteria belonging to the Bacteroidetes are represented by several* Bacteroides* species, including* B. acidifaciens*,* B. sartorii*, and* B. uniformis*. The remainder of the commensal bacteria, accounting for less than 10% of the total population, belongs to the phyla Proteobacteria, Fusobacteria, Actinobacteria, Verrucomicrobia, Cyanobacteria, and TM7. These bacteria are capable of successfully competing with members of the Firmicutes and Bacteroidetes in a strict anaerobic environment, such as the colon [26, 27].

The importance of the intestinal microbiota to host health is highlighted by alterations in community composition in metabolic and inflammatory diseases, such as obesity and IBD [5, 6]. The impact of the gut microflora on aGVHD has been shown to be highly significant (Table 1). Earlier studies in mice showed that symptoms of aGVHD could be reduced with antibiotics and transplantation in germ-free conditions [28, 29]. One recent study showed how intestinal microbiota influences aGVHD progression following allogeneic bone marrow transplantation (allo-BMT) [19]. Analysis of the microbiota composition indicated that recipient mice that developed aGVHD had a dramatic loss of bacterial diversity and a distinct composition compared with recipient mice that did not develop aGVHD. In mouse models of aGVHD, there is marked expansion of* Lactobacillus johnsonii* and a decrease in both Clostridiales and in other members of the phylum Firmicutes in the ileum. To determine the connection between* L. johnsonii* and aGVHD, the recipient mice were treated with antibiotics and then gavaged with* L. johnsonii* prior to allo-BMT. Antibiotic-treated mice showed a loss of Clostridiales and an emergence of* Enterococcus* spp., which was associated with exacerbated aGVHD [30]. In contrast, mice reintroduced with* L. johnsonii* showed no expansion of* Enterococcus* spp. and did not experience increased aGVHD lethality or pathology. These results suggest that* L. johnsonii* may reduce aGVHD severity by preventing the expansion of* Enterococcus* spp. A clinical research study elucidated variations in the intestinal microbiota of 31 patients receiving allogeneic HSCT [31]. The result of metagenomic analysis showed that patients had a predominance of commensal bacteria at the time of admission. After transplantation, a relative shift toward Enterococci was observed, and this shift was particularly prominent in patients that developed subsequently active gastrointestinal aGVHD. Another clinical study revealed that the diversity of intestinal microbiota at engraftment is an independent predictor of mortality in allo-HSCT recipients. Mortality outcomes were significantly worse in patients with lower intestinal diversity [32].

## 3. Intestinal Barrier Damage and LPS-Induced Septicemia during aGVHD 

Scientists have described several requirements for aGVHD development. First, the graft infused into the patient must contain immunological cells, such as mature T cells. Second, the recipient must be immunocompromised and cannot reject cells from the donor. Third, there are immunologic disparities between the recipient and the donor tissue cells. It is accepted that the development of aGVHD can be conceptualized as a three-stage process [15, 36]. First, antigen-presenting cells (APCs) are activated. Underlying diseases, previously administered therapies and the HSCT conditioning regimen can damage host tissues, resulting in the production of “danger signals,” such as proinflammatory cytokines, chemokines, MHC antigens, and costimulatory molecules on host APCs. Second, donor T cells proliferate and differentiate in response to activated host APCs. Additional inflammatory cytokines are then released. Finally, a complex cascade is triggered. Both cellular mediators, such as cytotoxic lymphocytes (CTLs) and NK cells, and inflammatory factors, such as tumor necrosis factor alpha (TNF-*α*), interferon gamma (IFN-*γ*), and interleukin-1 (IL-1), can cause the destruction of the target tissue, typically the skin, liver, and gut.

**Figure 1:**
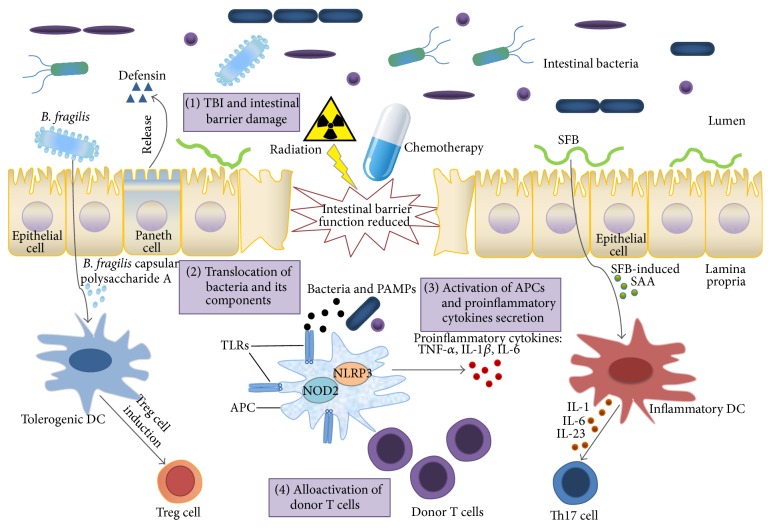
Schematic of the role of microbiota in graft-versus-host disease in the gut. Under normal conditions, the intestinal epithelial surface maintains an intact barrier function that prevents bacterial invasion into deeper host tissues. Paneth cells secrete several microbicidal proteins, including *α*-defensins, which selectively kill pathogenic bacteria.* Bacteroides fragilis*-derived TLR capsular polysaccharide A can promote the induction of Treg cells. Segmented filamentous bacteria (SFB) induce production of serum amyloid A (SAA) in the gut, and SAA acts on dendritic cells (DCs) to promote Th17 cell differentiation. After total body irradiation (TBI) and chemotherapy as part of the conditioning regimen, the integrity of the intestinal surface is decreased. Intestinal bacteria and their components (pathogen-associated molecular patterns, PAMPs) translocate to the lamina propria and are recognized by Toll-like receptors (TLRs) in host antigen-presenting cells (APCs). Activated APCs secrete proinflammatory cytokines and prime donor T cells, which aggravate acute GVHD.

It has been proposed that the GI tract may be particularly critical at the initial stage. Both total body irradiation (TBI) and high-intensive chemotherapy as part of the conditioning regimen can reduce or eliminate tumor load and cause sufficient immunosuppression to prevent graft rejection. However, such treatments may also stimulate host tissues to secrete inflammatory cytokines, such as TNF-*α* and IL-1, and their direct influence on epithelial cells of the GI tract allows for the translocation of intestinal microbes and their by-products, including lipopolysaccharide (LPS) and peptidoglycan, into the systemic circulation (Figure 1).

The symbiotic nature of the intestinal host-microbial relationship is dependent on limiting bacterial penetration of host tissues. Controlling bacterial interactions with the intestinal surface is an important strategy for minimizing bacterial translocation [37]. The intestinal epithelial surface is the primary interface between the gut bacteria and deeper tissues. Given the enormous numbers of commensal microbes and the persistent invasion of pathogens, it is important that the host immune system monitors and regulates microbial interactions with the intestinal surface [38].

The intestinal surface maintains an intact barrier through formation of complex protein-protein networks that firmly join together via tight junctions [39]. In HSCT, both TBI and chemotherapy can cause intestinal mucosa damage. Following this, intestinal bacteria may translocate into deeper tissues from the damaged GI tract and cause infection and septicemia [40]. In a mouse model of aGVHD, serious impairment of intestinal barrier function in the jejunum was detected, with increased permeability and morphological changes owing to both decreased protein expression and altered localization of the tight junction protein occluding [41]. There is a significant relationship between gut microbiota and intestinal radiosensitivity. In a mouse model of TBI-BMT, germ-free mice were markedly resistant to lethal radiation enteritis [42]. Several commensal bacteria or their components have the ability to protect intestinal mucosal tissue from irradiation damage. Bacteria-derived flagellin pretreatment protected mice from radiation-induced intestinal mucosal injury and apoptosis via a Toll-like receptor 5 (TLR5)-dependent mechanism [43]. In a human trial, patients taking the probiotic mixture VSL#3 (a mixture of eight probiotic strains) experienced radiation-induced diarrhea less frequently than patients taking a placebo in a double-blinded study [44]. Another study showed that a* Lactobacillus rhamnosus *GG- (LGG-) derived soluble protein, p40, ameliorates intestinal injury and colitis, reduces apoptosis, and preserves barrier function by transactivation of the EGF receptor (EGFR) in intestinal epithelial cells [45].

The Paneth cell is an intestinal epithelial cell that plays a key role in limiting bacterial penetration into host tissues. Paneth cells secrete the majority of antimicrobial proteins produced by the small intestine. These cells have a much longer half-life than other cells found in the small intestine [46]. Paneth cells can secrete a number of microbicidal proteins, including *α*-defensins, which selectively kill pathogens, while preserving commensals. When Paneth cells sense bacterial signals, they react by discharging their microbicidal granule contents into the gut lumen [47]. Therefore, Paneth cells are critical to the immune response to pathogens and the maintenance of a noninflammatory commensal flora in the small intestine. Current studies show that Paneth cells are targeted by aGVHD, resulting in a substantial loss of Paneth cells in the intestine and marked reduction in the expression of *α*-defensins in recipients with aGVHD. Restriction fragment length polymorphism (RFLP) of intestinal microbial communities showed loss of physiologic diversity among the microbiota and the overwhelming expansion of a rare bacterium. 16S rRNA gene sequencing demonstrated that this peak was almost certainly due to the presence of* Escherichia coli* in the intestine of recipients suffering from aGVHD [18]. Another clinical study showed that enumeration of duodenal Paneth cells is a readily available index of disease severity that provides important information regarding aGVHD prognosis [48].

aGVHD and related infections are major obstacles to HSCT. Septicemia is the most life-threatening infection following allogeneic HSCT, and gram-negative bacteria are the most dominant pathogens of septicemia. aGVHD is regarded as one of the major predisposing factors for the development of septicemia. LPS can enter the circulation through the impaired mucosal barrier after the conditioning regimen. In experimental studies, the proinflammatory potency of LPS varies from bacterial species to species. Probiotic microorganisms have been shown to alter the composition of the intestinal microflora and thereby mediate anti-inflammatory effects. Modifying the intestinal microbiota by oral administration of LGG before and after transplantation resulted in improved survival and reduced aGVHD. Furthermore, subculture of mesenteric lymph nodes revealed a reduced translocation of enteric bacteria [17].

## 4. Connections among Intestinal Microbiota, Innate Immune Receptors, and T Cell Differentiation during aGVHD

Connections between pathogen-associated molecular patterns (PAMPs) and pathogen recognition receptors (PRRs) control adaptive immune responses in inflammatory disorders, including aGVHD. Intestinal bacteria and their components are recognized by PRRs in antigen-presenting cells (APCs). The stimulation of PRRs leads to transcription of inflammatory genes and upregulation of proinflammatory cytokines and class II and costimulatory molecules, resulting in local tissue inflammation and migration of leukocytes [49].

Intestinal mucosal surfaces are rich in resident innate immune cells, such as macrophages and dendritic cells (DCs). Signaling through PRRs regulates the activity of DCs, leading to phagocytosis, chemokine receptor expression, cytokine secretion and migration from peripheral tissue to draining lymph nodes, and antigen presentation (Figure 1). Several TLRs have been described to recognize different PAMPs, including gram-positive bacteria-derived lipoproteins (TLR2), gram-negative bacteria-derived LPS (TLR4), bacterial flagellin (TLR5), RNA (TLR3, TLR7, and TLR8), cytosine-phosphorothioate-guanine (CpG) DNA (TLR9), profilin (TLR11, TLR12), and bacterial 23S ribosomal RNA (TLR13) [50, 51]. TLR downstream signaling activates a complex signaling cascade, eventually leading to host resistance against pathogens by increased production of cytokines, chemokines, adhesion molecules, and antimicrobial peptides, as well as to enhanced antigen presentation by APCs. PAMPs are recognized not only by TLRs but also by nucleotide-binding oligomerization domain- (NOD-) like receptors (NLRs). NLRs include proteins such as NALPs, NOD1, and NOD2, which are involved in the secretion of inflammatory cytokines, such as IL-1*α* and IL-18 [52].

As recognized molecular patterns of invading and resident microbes, TLRs are fundamental to controlling intestinal tissue homeostasis in studies that involve intestinal epithelial cell damage. In experimental aGVHD, TLRs appear to have a significant role in disease outcomes. In models of HSCT, TLR9^−/−^ transplant recipient mice have enhanced survival compared to wild type mice [53]. However, the role of TLR4 in aGVHD is still unclear, with conflicting findings in different studies [54, 55]. Evaluation of the expression of TLRs on T lymphocytes and monocytes in 34 patients showed that levels of TLR5 on monocytes and T lymphocytes are positively correlated with aGVHD, whereas levels of TLR1 and TLR9 are negative predictors [56].

The pathophysiology of aGVHD is a multistep process that eventually results in T helper 1-driven (Th1-driven) tissue damage. Recently, increasing evidence indicates the involvement of T helper 17 (Th17) and regulatory T cells (Tregs) in aGVHD pathogenesis [57]. One clinical study showed that* in situ* quantification of the Th17/Treg ratio was a specific marker of human aGVHD [58]. Murine experimental studies provide inconsistent results on the role of Th17 in aGVHD [59–61]. In contrast, Treg contributes to tolerance acquisition to donor antigen in solid organ transplantation and protects the development of fatal aGVHD in murine model [62].

The homeostasis of steady-state mucosal T cell subsets is controlled by signals from various components of the intestinal microbiota [63, 64]. Treg and Th17 cells are the most abundant lamina propria CD4^+^ T cell subsets at steady state. Tregs are crucial in inhibiting excessive inflammatory responses toward intestinal bacteria [65, 66]. Th17 cells are characterized by the production of IL-17 and other effector cytokines, such as IL-17F and IL-22. Th17 cell-derived cytokines function as important activators of innate immune mechanisms, such as recruitment of neutrophils and induction of antimicrobial peptide production from epithelial cells. Th17 cells also play crucial roles in mucosal defense against bacteria and fungi [67]. Treg and Th17 cells have reciprocal functions in regulation of intestinal microbiota. Treg and Th17 cell numbers in the gut are controlled by signals from different species of the commensal microbiota [68, 69]. Colonic Treg cells are induced by bacteria belonging to group IV and XIVa Clostridia, and small intestinal Th17 cells are induced by segmented filamentous bacteria (SFB) [70–72].

## 5. Probiotics and Their Potential Application in the Treatment of aGVHD

Patients undergoing allo-HSCT have a substantially increased risk of bacterial, fungal, and viral infection. Multiple approaches to decrease the risk of infections after HSCT have been explored, including laminar airflow housing, the use of antibiotics, and prophylactic antiviral and antifungal therapies [73]. Antibiotic resistance in pathogenic bacteria has been an increasing threat to human health during the last decade, and it is widely accepted that the antibiotic resistance development and spread in microbes can be largely attributed to the abuse and misuse of antibiotics [74]. In addition, breakdown of the normal microbial community by antibiotic use increases the risk of pathogen infection and the overgrowth of harmful disease.* Clostridium difficile* infection (CDI) is the most common cause of severe diarrhea and occurs with increased frequency after broad-spectrum antibiotic treatment [75, 76]. Results from a retrospective study showed that CDI is strongly associated with aGVHD and increased nonrelapse mortality in allo-HSCT patients [77]. In recent years, fecal microbiota transplantation (FMT) has emerged as an efficacious method for the treatment of CDI [78, 79]. FMT refers to infusion of a fecal suspension from a healthy individual into the GI tract of a patient to restore healthy intestinal microbiota and cure disease. However, there is risk associated with FMT. This is because fecal suspension contains harmful bacteria, viruses, and parasites. An alternative solution to this problem is to screen some beneficial bacteria from stool and only infuse these bacteria as a cocktail into the GI tract [80, 81].

Probiotics are live microorganisms, which when administered in adequate amounts confer a health benefit to the host. Probiotic strains are derived from fermented foods, beneficial commensals, or the environment. A broad range of consumer products containing probiotic microbes is currently available. Various animal and human studies have demonstrated that some probiotic strains can successfully modify the mucosal immune response due to species and strain specificity of the probiotics [82, 83]. The most commonly used probiotics are* Bifidobacterium*,* Bacillus*, and the lactic acid bacteria (LAB), including genus* Lactobacillus* and* Streptococcus* [84].* Lactobacillus rhamnosus *ATCC 53103, isolated from a healthy human intestinal commensal, is one of the most widely used and well-documented probiotic strains [17, 45, 85–87]. Some of the beneficial impacts of probiotics have been scientifically documented, and three main categories of probiotics contributing to host health have been described [83, 88, 89]. First, certain probiotics can exclude or inhibit pathogens. Second, probiotics can enhance the function of the intestinal epithelial barrier by modulating various signaling pathways, inducing mucus and antimicrobial peptide production, enhancing tight junction functioning, and preventing apoptosis. Third, probiotics can modulate host immune responses, resulting in both local and systemic effects.

The rationale for using probiotics to treat gastrointestinal disorders is supported by several clinical studies. In one study, ingestion of* Bifidobacterium infantis *35624 was shown to alleviate symptoms of irritable bowel syndrome (IBS) associated with normalization of the ratio of anti-inflammatory to proinflammatory cytokines [90]. To assess the effects of probiotics in diarrhea, a meta-analysis that included 63 studies (*n* = 8014) was carried out; the results showed that probiotic strains appear to be safe and have clear beneficial effects in shortening the duration and reducing stool frequency in acute infectious diarrhea [91]. Animal models indicate the close connection between gut microbiota and IBD, and several kinds of probiotics were evaluated in human clinical trials [92, 93].* Escherichia coli *Nissle 1917 shows efficacy and safety in maintaining a remission equivalent in patients with ulcerative colitis [94]. Another clinical study evaluated the impact of VSL#3 on maintenance of remission. The once daily high dose probiotic VSL#3 is effective in maintaining antibiotic-introduced remission for at least one year in patients with recurrent or refractory pouchitis [95].

There are several bottlenecks that need to be overcome before probiotics can be applied in clinical treatment. One important limitation is the delivery of viable cells to the lower GI tract without a significant loss of cell viability and metabolic features through the harsh conditions of the stomach [96]. In addition, compared to animal models, a major challenge for understanding the interactions between intestinal microbiota and human hosts is the heterogeneity of microbial composition that can colonize the intestine. Different components of microbiota can have very different effects on the host, and the composition of microbial communities can be influenced by a variety of factors, including diet, antibiotic therapy, and environmental exposure to microbes [97, 98].

## 6. Conclusions and Future Perspectives 

An increasing number of studies have identified an important interaction between intestinal microbiota and mammalian host health and disease. It is clear that microbiota can affect disease progression in several experimental animal models. Recent evidence has demonstrated that specific species of intestinal bacteria appear to be specialized in their ability to induce particular immune cell subsets. Probiotic bacteria can modulate immune responses to promote health by altering the composition of intestinal microbiota. Manipulation of the intestinal microbiota through probiotics or their components holds great promise for the treatment of inflammatory diseases, including aGVHD.

Despite some notable successes from probiotics trials, there are numerous obstacles to overcome before probiotic strains can be utilized as aGVHD therapeutics. First, most of the probiotics are species or strain specific. Thus, they should be tested* in vitro* and then evaluated for their suitability and efficacy* in vivo*. Only a few probiotic strains have been tested in mouse aGVHD models to date. Hence, there remains a pressing need to screen for probiotic strains that display more remarkable therapeutic responses to aGVHD. Second, although changes in microbiota associated with aGVHD progression are apparent, it is unknown which exact components of the microbiota are responsible for aggravating or ameliorating aGVHD. Therefore, monitoring intestinal microbiota changes of patients before and after aGVHD is of vital significance. Third, the association of intestinal microbiota with aGVHD has become evident. However, causality of aGVHD in response to probiotic-induced microbiota changes still has not been demonstrated. Along with research on screening for safe and effective probiotic strains moving towards increasing profundity, more attention should be focused on the immune mechanisms governing probiotic effects in aGVHD. Several creative therapeutic methods might originate from a better understanding of the relationship among probiotic strains, intestinal microbiota, and immune regulation during aGVHD.

## Figures and Tables

**Table 1:** Effect of the intestinal microbiota on GVHD.

Host	Outcome	Possible mechanisms	Refs.
Mouse	Oral administration of *Lactobacillus rhamnosus* GG (LGG) before and after transplantation results in improved survival and reduced aGVHD	Mice treated with LGG have a reduced translocation of enteric bacteria	[17]

Mouse	Loss of physiologic diversity among the intestinal microbiota and the overwhelming expansion of *Escherichia coli* which caused septicemia	Paneth cells are targeted by GVHD, resulting in marked reduction in the expression of *α*-defensins	[18]

Mouse	Loss of overall diversity of gut microbiota. Eliminating Lactobacillales from the mice before BMT aggravated GVHD, whereas reintroducing *Lactobacillus johnsonii* mediated significant protection against GVHD	*L. johnsonii* reduced GVHD severity by prevention of *Enterococcus *expansion	[19]

Mouse	Increased bacterial translocation and serum lipopolysaccharide (LPS) levels were detected after TBI	Neutrophil granulocytes recruited upon translocation of intestinal bacteria enhance GVHD via tissue damage	[33]

Mouse	The inflammatory responses in intestinal GVHD (iGVHD) were accompanied by gut flora shifts towards Enterobacteria, Enterococci, and Bacteroides/Prevotella spp.	iGVHD development is mediated by MyD88/TLR9-dependent bacterial sensing	[34]

Human	Successful total gastrointestinal decontamination (GID) of the graft recipient prevents moderate to severe acute GVHD	Prevention of intestinal microorganisms translocation	[35]

Human	After transplantation, a relative shift toward Enterococci was observed, especially in patients that developed subsequently or suffered from active GI GVHD	Early microbiome shifts may affect intestinal inflammation in the setting of allogeneic SCT	[31]

Human	Mortality outcomes were significantly worse in patients with lower intestinal tract bacterial diversity	Intestinal microbiota may be an important factor in the success or failure in allo-HSCT	[32]
